# Pemphigoid gestationis and preeclampsia in a donor-egg IVF pregnancy

**DOI:** 10.1097/JW9.0000000000000026

**Published:** 2022-07-12

**Authors:** Timothy L. Cowan, Mani Makhija, Dedee F. Murrell

**Affiliations:** Department of Dermatology, St George Hospital, Kogarah, New South Wales, Australia, Faculty of Medicine, University of New South Wales, New South Wales, Australia; Kossard Dermatopathologists, Macquarie Park, Sydney, New South Wales, Australia; Department of Dermatology, St George Hospital, Kogarah, New South Wales, Australia, Faculty of Medicine, University of New South Wales, New South Wales, Australia

**Keywords:** blistering, egg donation, IVF, pemphigoid gestationis, preeclampsia, pregnancy

**What is known about this subject in regard to women and their families?**

- There is no data prospectively collected on the link between in-vitro fertilisation with a donated egg and pemphigoid gestationis.
- One case report suggested that donor eggs may cause an allogeneic response.

**What is new from this article as messages for women and their families?**

- When seeing a dermatologist for a rash in pregnancy, it is important to consider other conditions of pregnancy such as preeclampsia.
- A donated egg in an IVF pregnancy may trigger pemphigoid gestationis.
- Pemphigoid gestationis does not always present with blisters.

## Dear Editors,

**Fig. 1.**
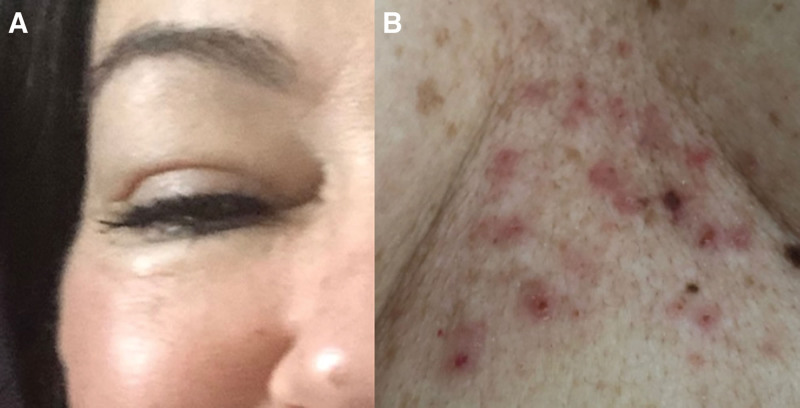
Photographs of clinical manifestations of pemphigoid gestationis. (A) Urticarial papules and erosions on chest. (B) Periorbital edema.

A 47-year-old Caucasian female underwent her second in-vitro fertilisation pregnancy with the same unrelated egg donor aged 29 and same father aged 44. Early in her pregnancy, she developed pregnancy-induced subclinical hypothyroidism with negative thyroid autoantibodies, normal T4, and a thyroid-stimulating hormone of 2.7, which is elevated compared with the normal range in the first trimester of pregnancy and was commenced on levothyroxine. In the second trimester of this pregnancy, she developed intensely pruritic erythematous patches on her chest (Fig. 1A), which then spread to her trunk and limbs, but spared her peri-umbilical region without obvious blistering. She was referred for investigation of her worsening pruritus despite regular antihistamines and topical steroids.

**Fig. 2.**
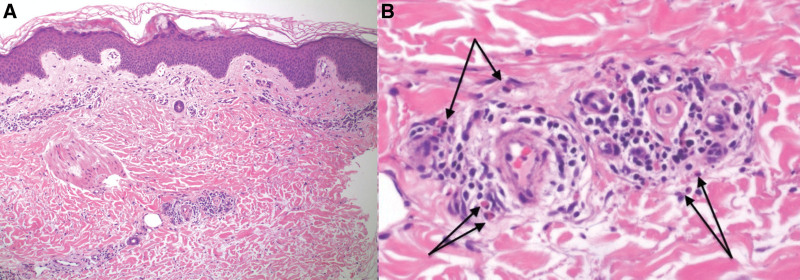
Histopathology of lesional skin biopsy. (A) Low power histopathology revealing eosinophils, neutrophils, and edema in the papillary dermis and perivascular inflammation. (B) High power with arrows highlighting eosinophils.

A lesional skin biopsy, stained with hematoxylin and eosin, revealed eosinophils, neutrophils, and edema in the papillary dermis and perivascular inflammation (Fig. 2). On direct immunofluorescence of perilesional skin, there was discontinuous n-serrated linear deposition of complement (C3) at the dermoepidermal junction. Bullous pemphigoid (BP) 180 antibodies were positive with a titer of 3.11 on serum enzyme-linked immunosorbent assay, with negative BP230 antibodies. Pemphigoid gestationis (PG) was diagnosed rather than polymorphic eruption of pregnancy due to her positive direct immunofluorescence and BP180 antibodies.

During follow-up, consultation to explain her diagnosis of PG, facial and peripheral edema was noted, and preeclampsia was considered. Her blood pressure was 162/95 with proteinuria, and so she was urgently admitted to the hospital. She had been closely monitored during her pregnancy due to her high-risk status from advanced maternal age, and this was the first evidence of preeclampsia during her antenatal care. Therefore, her manifestations of PG commenced approximately 16 weeks before her preeclampsia. There was no personal or family history of hypertension or preeclampsia in the egg donor or father; however, there was a maternal family history of hypertension in her father.

For severe preeclampsia, she was managed with labetalol, hydralazine, and intravenous magnesium sulfate but proceeded to have an emergency cesarean at 32 weeks resulting in a male infant with a birth weight of 1.58 kg, Appearance, Pulse, Grimace, Activity and Respiration 5, 7, and 9 at birth, 5 and 10 minutes. He remained in the neonatal intensive care unit and was discharged well at 9 weeks. By 1-week post-delivery, her PG lesions had completely resolved but she had ongoing positive BP180 antibodies at a reducing titer. By 4 weeks post-partum, her hypertension had resolved and anti-hypertensives had ceased.

PG is a rare autoimmune disease of pregnancy.^1^ There has been 1 reported case of PG coinciding with Haemolysis, Elevated Liver enzymes, and Low Platelet count syndrome,^2^ and a small number of case studies reporting association between PG and preeclampsia.^3,4^ Donated eggs have been proposed as an allogeneic trigger for PG through the generation of BP180 antibodies from abnormal expression of major histocompatibility complex class II molecules on chorionic villi. This may develop an augmented immune response to the entire fetal genome.^5^ In this case, the mechanism may underlie hypothyroidism, PG, and preeclampsia. There are no reported cases of a pregnancy complicated by new hypothyroidism, PG, and preeclampsia. This case is unique and may be the first immunogenic link between these 3 diagnoses.

This case also highlights the importance of holistic practice within dermatology. In any pregnant woman attending a dermatology clinic with a rash, it is prudent to consider other conditions of pregnancy that may be associated, such as preeclampsia, as in this case.

## Author contributions

All authors contributed to article writing and development. TLC and DFM were responsible for clinical information, and MM for histopathological analysis and results.

## Conflicts of interest

None.

## Funding

None.

## Study approval

N/A.

## Patient consent

Informed, written consent was received from all patients and confirmed to the journal pre-publication, stating that the patients gave consent for their photos and case history to be published.
